# Eosinophil–Basophil/Lymphocyte (EB/LR) and Eosinophil–Basophil–Platelet/Lymphocyte (EBP/LR) Ratios Could Serve as Useful Additional Markers for Assessing the Severity of Wasp Allergic Reactions

**DOI:** 10.3390/cells13211786

**Published:** 2024-10-28

**Authors:** Weronika Urbańska, Łukasz Szymański, Aneta Lewicka, Martyna Ciepielak, Karolina Kostrzeńska-Sęk, Andrzej Chciałowski, Sławomir Lewicki

**Affiliations:** 1 Department of Internal Medicine, Infectious Diseases and Allergology, Military Institute of Medicine, National Research Institute, Szaserów 128, 04-141 Warsaw, Poland; 2 Department of Molecular Biology, Institute of Genetics and Animal Biotechnology, Polish Academy of Sciences, Postępu 36A, 05-552 Magdalenka, Poland; 3 Military Centre of Preventive Medicine, Lesna 4D, Modlin, 05-100 Nowy Dwor Mazowiecki, Poland; 4 Department of Regenerative Medicine, Maria Sklodowska-Curie National Research Institute of Oncology, WK Roentgena 5, 02-781 Warsaw, Poland; 5 Institute of Outcomes Research, Maria Sklodowska-Curie Medical Academy, Pl. Żelaznej Bramy 10, 00-136 Warsaw, Poland

**Keywords:** wasp allergy, blood cells indexes, eosinophils and basophils ratio, EB/LR

## Abstract

Wasp venom allergy can trigger severe allergic reactions, and predicting these acute responses remains challenging. This study evaluates the utility of immune system indexes, particularly the eosinophil–basophil/lymphocyte (EB/LR) and eosinophil–basophil–platelet/lymphocyte (EBP/LR) ratios, in assessing the severity of allergic reactions in patients with wasp venom allergy. A total of 61 patients with confirmed wasp venom allergy were categorized according to the Mueller scale, which classifies the severity of allergic reactions. Blood samples were analyzed for total and specific IgE levels alongside a range of hematological and biochemical parameters. This study found significant differences in the EB/LR and EBP/LR indexes between patients with mild (Mueller I–II) and severe (Mueller III–IV) allergic reactions, with higher values indicating more severe responses. However, no significant differences were observed in other immune indexes, such as the platelet-to-lymphocyte ratio, neutrophil-to-lymphocyte ratio, systemic immune-inflammation index, and systemic inflammatory response index, as well as in additional blood parameters. These findings suggest that the EB/LR and EBP/LR ratios may serve as useful markers for predicting the severity of allergic reactions in patients with wasp venom allergy. This is the first study to establish such a link, although further research with larger cohorts is necessary to confirm these results and their potential application in clinical settings.

## 1. Introduction

Insect venom allergy is an exaggerated immune response triggered by an insect sting [1]. Allergic reactions are most commonly caused by venom from insects belonging to the order *Hymenoptera*, which is one of the largest insect orders, comprising approximately 200,000 species of bees, wasps, and ants [2]. In Europe, the majority of allergic reactions following insect stings are due to insects from the Apidae (bees) and *Vespidae* (wasps) families. Notably, the *Vespidae* family alone includes over 6000 species of wasps worldwide [3]. The chemical composition of wasp venom primarily includes enzymatic compounds, neurotoxins, and neuropeptides that specifically target the nervous system [4]. In some individuals, these venom components can also trigger an immune response, leading to allergic reactions [5].

Following an insect sting, a range of reactions may occur, including a normal local reaction, a large local reaction, or a systemic reaction, which can escalate to anaphylactic shock. In mild cases of allergy to *Hymenoptera* venom, symptoms typically include itching, urticaria, angioedema, gastrointestinal symptoms such as vomiting, abdominal pain, and diarrhea corresponding to grades I and II on Mueller’s scale, respectively. Severe systemic reactions are categorized as grade III, characterized by respiratory symptoms such as stridor, shortness of breath, cough, and bronchial obstruction, and grade IV, which involves cardiovascular symptoms like hypotension, tachycardia, arrhythmias, and cardiac arrest [6].

The prevalence of *Hymenoptera* venom allergy varies based on geographical location, occupation, and individual susceptibility. Globally, approximately 0.9% to 3.4% of children and 5.0% to 8.9% of adults may develop a systemic allergic reaction to a sting [7]. The risk is higher among beekeepers, outdoor workers, and individuals with a history of allergic reactions to insect stings [8]. In Europe, insect venom allergy is the most common cause of anaphylaxis in adults and the second most common cause in children, following food allergies. This condition primarily involves the venoms of wasps and honeybees [3].

According to European data, the incidence of systemic reactions to bee and wasp stings ranges from 0.3% to 8.9% in adults and up to 3.4% in children. The incidence is even higher among beekeepers, reaching up to 32%, with large local reactions occurring in 2.4% to 26.4% of the general population [9]. The estimated number of deaths resulting from sting reactions varies from 0.03 to 0.45 per million inhabitants per year [10]. Moreover, insect stings are responsible for approximately 50% of severe anaphylaxis cases in adults and about 20% in children [11]. The most effective way to prevent serious reactions following a wasp sting is to avoid areas where these insects are common or to undergo allergen immunotherapy. Immunotherapy prevents systemic reactions after subsequent stings and significantly improves patients’ quality of life. The effectiveness of desensitization to wasp venom is estimated to be greater than 90% and for bee venom, greater than 80% [12]. In Poland, fewer than 3000 people receive venom immunotherapy (VIT) each year. VIT is recommended only for patients who meet both clinical and immunological criteria: a history of severe generalized reactions to stings involving respiratory or circulatory symptoms (grades III–IV according to Mueller’s scale) and the presence of specific IgE (sIgE) antibodies against the venom of the insect responsible for the reaction, confirmed by positive skin test results or serum antibody assays [13]. However, both criteria have limitations: the clinical assessment of systemic reactions over time can be subjective, and IgE levels in the blood may decrease relatively quickly due to elimination from circulation and binding to tissue-resident mast cells [14]. Furthermore, according to guidelines from the European Academy of Allergy and Clinical Immunology (EAACI) and the American Academy of Allergy, Asthma & Immunology (AAAAI), VIT may also be considered for adults with moderate allergic symptoms (grade I–II according to Mueller), particularly in patients over 65 years of age with cardiovascular diseases and/or primary or secondary mast cell activation syndromes [12]. Given these complexities, the decision to use VIT is not always straightforward. As a result, ongoing research is focused on identifying new objective markers or indicators that can help guide treatment decisions more effectively.

The markers currently used, such as specific IgE, although valuable, do not provide comprehensive insights into the inflammatory processes, particularly the role of eosinophils, basophils, and mast cells, which are central to the allergic response. Mast cells and basophils are not routinely quantified in clinical practice, whereas eosinophils are readily available through a complete blood count. Consequently, blood-count-based markers, particularly those involving eosinophils, have the potential to fill this gap and provide valuable insights into the severity and management of venom allergies.

In recent years, numerous studies have been conducted to evaluate the usefulness of blood count parameters as prognostic indicators in various diseases. Complete blood count (CBC) analysis is a widely available and cost-effective test, and its interpretation can yield valuable clinical insights. Recently, new markers of inflammatory activity have been developed, including the neutrophil-to-lymphocyte ratio (NLR), platelet-to-lymphocyte ratio (PLR), eosinophil-to-lymphocyte ratio (ELR), monocyte-to-lymphocyte ratio (MLR), and red blood cell distribution width (RDW) [15,16,17]. It is well established that both allergic and inflammatory reactions can induce changes in blood morphology parameters. For instance, studies have shown that ELR is significantly elevated in pediatric patients with allergic rhinitis [18]. In recent years, markers such as NLR, PLR, and ELR have been utilized by researchers as prognostic indicators in the management of various inflammatory conditions, including atopic dermatitis (AD) [19,20,21] and asthma [22,23]. The assessment of blood count biomarkers in venom allergies presents a valuable opportunity for developing a simple, cost-effective, and rapid approach to evaluating allergic responses. These markers could complement established tools, such as specific IgE measurements, by offering additional insights into the immune mechanisms involved in allergic reactions. In particular, eosinophil-based ratios—such as the eosinophil-to-basophil/lymphocyte ratio (EB/LR) and the eosinophil–basophil–platelet-to-lymphocyte ratio (EBP/LR)—are of interest in this study. Their potential to serve as prognostic markers may enhance the ability to predict clinical outcomes and responses to venom immunotherapy. The integration of these markers into clinical practice could improve diagnostic precision and support more tailored treatment strategies for patients with venom allergies.

Given this background, the present study aims to evaluate the utility of these established immunological indices, such as NLR, PLR, and ELR, in assessing patients with wasp sting allergy. By focusing on eosinophil-based indices, this study intends to fill the current gap in predicting allergic responses and improve the selection process for venom immunotherapy.

## 2. Materials and Methods

### 2.1. Study Participants

This study was conducted as a retrospective analysis. This study involved 61 patients with confirmed hypersensitivity to wasp venom, recruited from the Department of Internal Medicine, Infectious Diseases and Allergology at the Military Institute of Medicine in Warsaw, Poland, as previously described [24,25]. The median age of the participants was 50 years, ranging from 20 to 70 years. Patients with contraindications to immunotherapy, such as active cancer, autoimmune diseases, or acquired immunodeficiency syndrome (AIDS), were excluded from this study, as were pregnant women. Wasp venom allergy was confirmed through a combination of patient history, skin prick tests, intradermal tests, and specific IgE (sIgE) measurements. Patients were classified according to the Mueller scale into two groups: those with Mueller scale grades III and IV and those with grades I and II [6]. A control group of healthy individuals was also included in this study. The Mueller scale I and II group comprised 18 patients, while the group with Mueller scale III and IV included 43 patients. The control group consisted of 46 healthy volunteers aged 26 to 63 years and was included in the analysis solely for the evaluation of blood indexes. This study was conducted in accordance with the resolution of the Bioethics Committee at the Military Institute of Medicine (No. 130/WIM/2018). All participants provided informed consent prior to their inclusion in this study.

### 2.2. IgE Analysis

Total IgE and specific IgE (sIgE) levels corresponding to wasp, bee, and hornet venoms were measured from serum samples, as previously described [24]. IgE and sIgE concentrations were determined using a fluorometric method with the UniCAP machine (HVD HOLDING AG, Warsaw, Poland). Specific antibodies against protein domains of wasp venom (rVes v1 and v5, rPol d5 [IU/mL]) and bee venom (rApi m1, m2, m3, m5, and m10) were also analyzed from serum obtained from peripheral blood. Blood samples were collected using the “clot” method, allowed to clot for 20 min, and then centrifuged at 2000× *g* for 20 min to separate the serum. The results are presented as the mean ± standard error of the mean (SEM).

### 2.3. Blood Parameters Analysis

Peripheral blood samples were collected in EDTA-containing tubes and immediately analyzed using the XN-1000 hematology analyzer (Sysmex Polska, Warsaw, Poland). The following parameters were measured: white blood cells (WBC [×10^9^/L]), red blood cells (RBC [×10^12^/L]), hemoglobin (HGB [g/dL]), hematocrit (HCT [%]), mean corpuscular volume (MCV [fL]), mean corpuscular hemoglobin (MCH [pg]), mean corpuscular hemoglobin concentration (MCHC [g/dL]), platelets (PLT [×10^9^/L]), mean platelet volume (MPV [fL]), lymphocytes ([×10^3^/µL] and [%]), neutrophils ([×10^3^/µL] and [%]), monocytes ([×10^3^/µL] and [%]), eosinophils ([×10^3^/µL] and [%]), and basophils ([×10^3^/µL] and [%]). The results are presented as the mean ± standard error of the mean (SEM).

### 2.4. Biochemical Analysis

Peripheral blood samples were collected in vacutainer tubes containing a gel facilitating clot activation (SST II Advance, BD Vacutainer, Becton Dickinson Polska, Warsaw, Poland) for the analysis of cholesterol, creatinine, urea, AST, and ALT. Additional samples were collected in sodium citrate tubes (BD Vacutainer Citrate Tubes, Becton Dickinson Polska, Warsaw, Poland) for fibrinogen analysis. Cholesterol, creatinine, urea, AST, and ALT levels were measured using the Cobas c 501 automatic biochemical analyzer (Roche Diagnostics, Warsaw, Poland) following the manufacturer’s standard procedures. Fibrinogen levels were analyzed using the Automatic Hemostasis Analyzer TOP 500 CTS (Werfen, Bedford, MA, USA) with HemosIL reagents, according to the manufacturer’s instructions. The results are presented as the mean ± standard error of the mean (SEM).

### 2.5. Immune System Parameters

Serum concentrations of interleukins (IL-1β, -2, -4, -5, -6, -7, -8, -9, -10, -12, -13, -15, -17A), TNF-α, IFN-γ, and TGF-β1 were analyzed as previously described, using the Bio-Plex Pro TGF-β 3-Plex Panel and the Bio-Plex Pro Human Cytokine Group I 27-Plex Panel on a Bio-Plex 200 system (Bio-Rad, Warsaw, Poland) [25].

### 2.6. Cells and Cytokines Indexes

The utility of various immunological indices was assessed to evaluate the potential for acute allergic symptoms following a wasp sting. Several established indices from the literature were utilized, including the platelet-to-lymphocyte ratio (PLR) [26], systemic immune-inflammation index (SII) [27], systemic inflammation response index (SIRI) [28], and neutrophil-to-lymphocyte ratio (NLR) [29], all calculated from hematological blood results:

- PLR (systemic inflammation index): Calculated from peripheral blood platelet (P) and lymphocyte (L) counts using the equation: PLR = P/L;
- SII: Calculated from peripheral blood platelet (P), neutrophil (N), and lymphocyte (L) counts using the equation: SII = P × N/L;
- SIRI: Calculated from peripheral blood monocyte (M), neutrophil (N), and lymphocyte (L) counts using the equation: SIRI = M × N/L;
- NLR: Calculated from peripheral blood neutrophil (N) and lymphocyte (L) counts using the equation NLR = N/L.

In addition to these established indexes, new indices were developed and evaluated that consider the specific immune system responses observed in allergic diseases. These new indices include the following calculations based on eosinophil (E) and basophil (B) counts:

- E/L: eosinophil-to-lymphocyte ratio (ELR);
- B/L: basophil-to-lymphocyte ratio (BLR);
- E × B/L: eosinophil × basophil-to-lymphocyte ratio (EB/LR);
- E × B × N/L: eosinophil × basophil × neutrophil-to-lymphocyte ratio;
- P × E/L: platelet × eosinophil-to-lymphocyte ratio;
- P × B/L: platelet × basophil-to-lymphocyte ratio;
- E × B × P/L: eosinophil × basophil × platelet-to-lymphocyte ratio (EBP/LR);
- N × M × E/L: neutrophil × monocyte × eosinophil-to-lymphocyte ratio;
- N × M × E × B/L: neutrophil × monocyte × eosinophil × basophil-to-lymphocyte ratio.

Furthermore, based on the cytokine study results, indexes for effector cytokines in the serum were calculated, corresponding to specific immune pathways: Th1 (IFN-γ × TNF-α), Th2 (IL-4 × IL-5 × IL-13), Th9 (IL-9), Th17 (IL-17), and Treg (IL-10 × TGF-β1), as described by Lee et al. 2021 [30]. The following calculations were performed:

- Th1/lymphocytes, Th2/lymphocytes, Th9/lymphocytes, Th17/lymphocytes, (Treg/lymphocytes)/10^3^;
- Th1/Th2, Th1/Th9, Th1/Th17, (Th1/Treg) × 10^3^;
- Th2/Th9, Th2/Th17, (Th2/Treg) × 10^3^;
- Th9/Th17, (Th9/Treg) × 10^3^, (Th17/Treg) × 10^3^.

### 2.7. Statistical Analysis

All results are presented as the mean ± SEM. Data distribution was assessed using the Shapiro–Wilk test. A *T*-test was applied to normally distributed data, while the Mann–Whitney test was used for data that did not follow a normal distribution. For multivariable analysis, the Kruskal–Wallis test was used. Spearman correlations were calculated for all parameters under investigation. Statistical significance was defined as *p* < 0.05. Statistical analyses and Receiver Operating Characteristic (ROC) curves for EB/LR and EBP/LR were performed using GraphPad Prism software (version 9.4.1; GraphPad Software, Inc., La Jolla, CA, USA).

Furthermore, correlations between EB/LR and EBP/LR markers and serum IgE levels were evaluated across patient groups.

## 3. Results

Only patients who met the inclusion and exclusion criteria were included in this study. Group assignment was based on the severity of the inflammatory reaction following the wasp sting.

### 3.1. IgE Analysis

**Figure 1:**
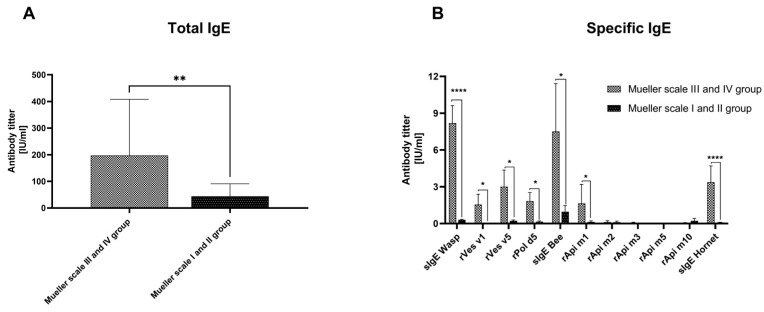
Total (**A**) and specific IgE (**B**) concentrations were determined in the serum of patients from the Mueller scale III and IV group and Mueller scale I and II group (Mueller scale I and II). Results are presented as mean ± SEM. * *p* < 0.05; ** *p* < 0.01; **** *p* < 0.0001.

Patients in the Mueller scale III and IV group exhibited significantly higher total IgE concentrations—approximately five times higher (*p* < 0.01)—in their serum compared to those in the Mueller scale I and II group. These elevated total IgE levels in the Mueller scale III and IV group were associated with markedly higher levels of specific IgE (sIgE) antibodies against wasp venom (about 30 times higher, *p* < 0.0001), as well as against bee venom (about eight times higher, *p* < 0.05) and hornet venom (about 35 times higher, *p* < 0.0001) compared to the Mueller scale I and II group. Additionally, specific antibodies against the wasp venom allergens rVes v1, rVes v5, and rPol d5 were significantly higher in the Mueller scale III and IV group than in the Mueller scale I and II group (*p* < 0.05) (Figure 1).

### 3.2. Blood Analysis

No significant differences were observed in the number and parameters of red blood cells and platelets between the Mueller scale III and IV and Mueller scale I and II groups. Similarly, immune system cell counts remained unchanged between the groups, except for the percentage of neutrophils, which was slightly higher in the Mueller scale I and II group than in the Mueller scale III and IV group (approximately 12%, *p* < 0.05, Table 1).

### 3.3. Biochemical Analysis

**Figure 2:**
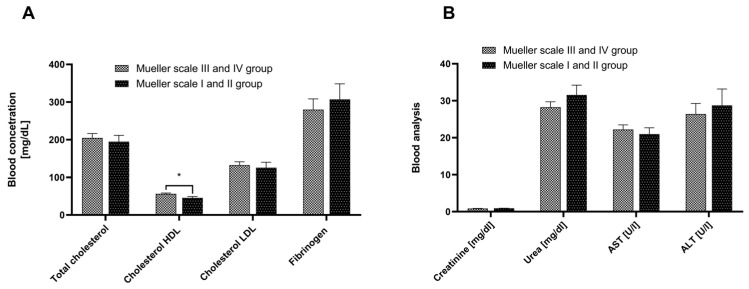
The concentration of cholesterol, fibrinogen (**A**), creatinine, urea, and activity of aminotransferases AST and ALT (**B**) determined in the serum of patients from the Mueller scale III and IV group and Mueller scale I and II group. Results are presented as mean ± SEM. * *p* < 0.05.

Participants in both the Mueller scale III and IV and Mueller scale I and II groups did not exhibit any significant deviations from the norm in terms of total cholesterol and LDL concentrations. However, the HDL level was slightly lower in the Mueller scale I and II group compared to the Mueller scale III and IV group (approximately 20% lower, *p* < 0.05). Both groups showed normal kidney function, with creatinine and urea levels within the standard range, and normal liver function, with AST and ALT levels also within the norm. Fibrinogen levels were comparable between the two groups. The data described above are presented in Figure 2.

### 3.4. Index Analysis

**Figure 3:**
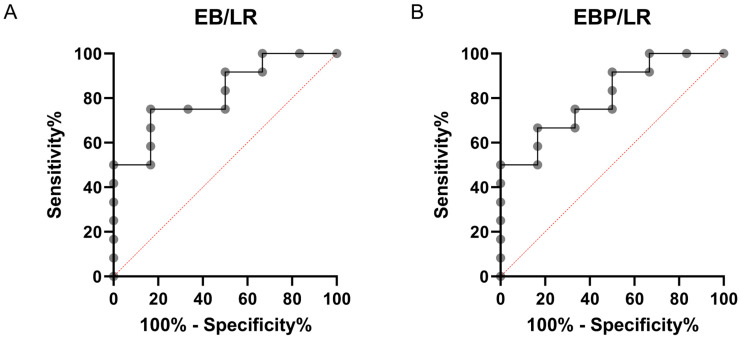
Receiver Operating Characteristic (ROC) curves for EB/LR (**A**) and (**B**) EBP/LR indexes. Panel A shows the ROC curve for EB/LR with an AUC of 0.8194 (95% CI: 0.6188–1.000, *p* = 0.0312). Panel B presents the ROC curve for EBP/LR, with an AUC of 0.8056 (95% CI: 0.5984–1.000, *p* = 0.0394).

No significant changes were observed in the commonly used immunological indexes from the literature, such as the systemic inflammation index, systemic immune-inflammation index, systemic inflammation response index, or neutrophil-to-lymphocyte ratio. Additionally, most other indices, including those involving cells of the innate immune system that play a role in the effector response in allergy, did not differ between the studied groups. The exception was the indexes involving eosinophil and basophil counts, with or without platelets, divided by the number of lymphocytes. In the calculation of the E × B/L (EB/LR) index, the average value in the Mueller scale I and II group was approximately twice as high as in the Mueller scale III and IV group (*p* < 0.05), and this difference persisted when platelet counts were included in the analysis (E × B × P/L, *p* < 0.05) (Table 2). There was no correlation between the level of IgE and EB/LR or EBP/LR ratio in the Mueller scale III and IV (r^2^ = 0.029, *p* = 0.95; r^2^ = 0.027, *p* = 0.98) and in the Mueller scale I and II group (r^2^ = −0.09, *p* = 0.79; r^2^ = 0.11, *p* = 0.73). The calculations of cytokine indexes did not reveal any significant differences between the groups (Table 3). However, Receiver Operating Characteristic (ROC) analysis supported the diagnostic usefulness of both the EB/LR and EBP/LR indexes, with area under the ROC curve (AUC) values of 0.8194 and 0.8056, respectively. These AUC values indicate good discriminative ability, exceeding the threshold of 0.8, which is considered indicative of reliable diagnostic performance [31]. The EB/LR index showed slightly better discriminative ability than the EBP/LR index, reinforcing their potential utility as diagnostic markers, despite the lack of correlation with IgE levels (Figure 3).

## 4. Discussion

In this study, two groups of patients with wasp sting allergies were compared based on their classification according to the Mueller scale: a Mueller scale I and II group and a Mueller scale III and IV group. The focus was on evaluating immunological parameters within a relatively small patient cohort. To minimize confounding factors, only patients who met the inclusion criteria were included in the analysis. Additionally, standard hematological and biochemical parameters were assessed for all patients. Comparisons between the Mueller scale I and II and Mueller scale III and IV groups revealed no significant differences in red blood cell (RBC), white blood cell (WBC), or platelet (PLT) counts, except for a slight increase in neutrophils in the Mueller scale I and II group. An increase in neutrophils in the blood is typically associated with infections, inflammation, or neoplastic processes. This study excluded patients with clinical signs of infection, cancer, or active inflammation. The normal range for absolute neutrophil counts in healthy adults is between 2.5 and 7.0 × 10³ cells/μL [32]. The neutrophil counts observed in our study, which varied between the groups, fall within this normal range for healthy individuals. As such, these differences are unlikely to have clinical significance and should not impact the results of the calculated parameters [33]. Biochemical analyses of liver and kidney function were also similar between the groups. The only notable difference was a slight variation in HDL levels, while LDL and total cholesterol levels showed no significant differences between the groups. These analyses were conducted to ensure that underlying organ damage or dysfunction did not confound the study results. Immunological status is influenced not only by infections but also by the overall health of organs and tissues, making it essential to assess biochemical parameters related to lipidemia, renal function, and liver function. Given the wide age range of participants (20 to 70 years, with a median age of 50), these tests helped to rule out significant organ dysfunction that could affect the immune response or inflammatory markers being studied. Although these parameters were not the primary focus, ensuring normal liver and kidney function was crucial for maintaining the integrity and reliability of the findings.

In an effort to identify parameters that could quickly and accurately predict an individual’s reaction to wasp venom allergy, we decided to examine IgE levels. Our analysis revealed that patients with lower grades on the Mueller scale had correspondingly lower levels of total IgE and specific wasp IgE in their serum. However, the relationship between venom-specific IgE levels and the severity of anaphylaxis is inconsistent across studies. For example, Day et al. found a correlation between the severity of anaphylaxis and the level of venom-specific IgE [34]. Similarly, Hollstein et al. observed significantly lower levels of venom-specific IgE in patients undergoing allergen immunotherapy (AIT) when comparing those in grade I to grade IV on the Mueller scale [35]. In contrast, Wilson et al. and Reisman and DeMasi reported no correlation between venom-specific IgE levels and the incidence of systemic reactions [36,37]. Kopač et al. also found that bee venom-specific IgE levels did not correlate with the occurrence of serious adverse events during specific immunotherapy (SIT) [38]. Nittner-Marszalska et al. further showed no relationship between skin test result size, FAST test class, and the severity of sting reactions classified by the Mueller scale [39]. Warrington confirmed these findings, reporting no significant correlation between venom-specific IgE levels or skin test reactivity and the severity of clinical reactions in venom-allergic patients [40]. Interestingly, Sturm et al. observed that in patients with mild reactions to *Hymenoptera* stings, high levels of total IgE were present, which decreased as the severity of the reaction increased according to the Mueller scale. They concluded that elevated total IgE levels often accompanied grade I and II reactions, suggesting a potential protective role against the development of more severe grade III reactions [41]. Additionally, a prospective analysis by Van der Linden et al. involving 138 patients with a history of anaphylaxis after an insect sting found no relationship between the severity of the reaction after a sting challenge and the levels of specific IgE, IgG4 in plasma, or skin test results [42]. Taken together, these data suggest that predicting the severity of an allergic reaction to a wasp sting based solely on IgE levels is challenging and remains an area of ongoing research.

Total IgE levels in blood can reflect an allergy to wasp venom, but they may also indicate an additional, undiagnosed allergic condition. For patients who are unable to identify the specific venom to which they are allergic, diagnostics based on allergen components, known as component-resolved diagnostics (CRD), have been employed [43]. Common recombinant allergens specific to bee venom include phospholipase A2 (rApi m 1), acid phosphatase (rApi m 3), mellitin (Api m 4), and icarapin (rApi m 10). In vespid venoms, the key allergens are phospholipase A1 (rVes v 1) and antigen 5 (rVes v 5). Additionally, there are homologous allergens, referred to as cross-reactants, which share a high degree of sequence identity between bee and wasp venoms. These include hyaluronidases (Api m 2 and Ves v 2), dipeptidyl peptidases IV (Api m 5 and Ves v 3), and vitellogenins (Api m 12 and Ves v 6) [44]. In our research, the levels of specific IgE varied between the Mueller scale III and IV and Mueller scale I and II groups, with the Mueller scale III and IV group generally exhibiting higher IgE levels for wasp venom components compared to the Mueller scale I and II group. Similar findings were reported by Kai et al., who demonstrated that the severity of allergic reactions in patients with venom allergies directly correlates with the specific IgE activity (sIgE/tIgE ratio) [45]. Furthermore, Hamilton et al. found that the sIgE/tIgE ratio can help differentiate true venom allergy from cross-reactivity, with a ratio exceeding 4% in 54% of patients with *Hymenoptera* venom allergy [46]. However, there are two significant limitations to the analysis of wasp venom components. First, this analysis is not yet recommended in the diagnostic guidelines for wasp venom allergy and is relatively costly. Second, and more importantly, it does not reliably predict potential reactions to wasp stings in all patients. In our study, some patients in the Mueller scale III and IV group exhibited low levels of IgE components, while some individuals in the Mueller scale I and II group had relatively high IgE levels. Similarly, Gattinger et al. observed severe anaphylactic reactions in patients with low IgE levels (<1 ISU and <2 kUA/L) for the key recombinant allergens Api m 1 and/or Ves v 5 [47].

The predictive utility of parameters such as PLR, NLR, SII, and SIRI for acute allergic responses to wasp stings remains uncertain. These markers have been widely used in diagnosing inflammatory diseases of bacterial–viral origin [26], immune-related conditions [23], and diseases associated with tissue damage [48]. However, their application in allergic diseases is less established. Some studies have linked increased NLR with chronic inflammation and conditions like asthma [49], allergic rhinitis [50], and psoriasis [51]. For instance, Jiang and Ma found higher NLR and PLR ratios in atopic dermatitis patients compared to healthy controls, with these ratios correlating with disease severity [19]. Consistent with these findings, our study demonstrates that while PLR and NLR indices can distinguish allergic patients from healthy individuals, they are not effective in differentiating between Mueller grade groups. Conditions like atopic dermatitis or psoriasis involve chronic inflammation, while wasp venom allergy primarily triggers acute reactions. Similarly, the SII and SIRI indexes, commonly used as prognostic markers in chronic inflammatory conditions such as type 2 diabetic retinopathy [51] and coronary artery disease [52], showed no significant differences between the Mueller scale groups in our study. Although these indexes have been effective in other immunological disorders, such as psoriasis [53], rheumatoid arthritis [54], and asthma [55], their relevance in acute allergic reactions, particularly in wasp sting allergy, remains unproven. Our findings suggest that SII and SIRI may not be reliable prognostic tools for acute allergic reactions to wasp stings. We found significant differences in the EB/LR (eosinophil–basophil/lymphocyte) and EBP/LR (eosinophil–basophil–platelet/lymphocyte) indexes, which effectively distinguished between the two groups of allergic patients in our study. Patients diagnosed at the III-IV stages of the Mueller scale exhibited approximately twice as high levels of indexes mentioned above levels compared to those diagnosed at the I-II stages. This is the first study to demonstrate an association between the EB/LR and EBP/LR indexes and the prognosis of acute allergic reactions following a wasp sting. The usefulness of these calculations has already been explored in other allergic diseases. Some studies have shown that the eosinophil and/or basophil to lymphocyte indexes are potential prognostic factors for atopic dermatitis [21,56]. Similarly, in allergic rhinitis, the EB/LR ratio has been found to distinguish between mild and severe cases of the condition [18]. Interestingly, we did not observe significant differences between the I-II and III-IV Mueller groups in the E/L (eosinophil/lymphocyte) or B/L (basophil/lymphocyte) ratios, suggesting that only the combined eosinophil and basophil counts relative to lymphocytes were able to differentiate the studied groups. Other blood parameters, such as monocytes or neutrophils, whether considered alone or in combination with eosinophils, basophils, or platelets, did not differ significantly between the groups, nor did their combination with cytokine levels in the blood (Table 3).

The authors recognize that this study is not without limitations. The cohort was relatively small, and this study could be considered preliminary. Nevertheless, our findings indicate that the EB/LR and EBP/LR indexes were significantly elevated only in the more severe cases (Mueller grades III-IV). To establish the broader applicability of these results, further analyses should be conducted on a larger patient group. Future research is necessary to confirm the prognostic utility of the EB/LR and EBP/LR indexes in allergic reactions to wasp stings.

## 5. Conclusions

In conclusion, our study is the first to associate the severity of the body’s reactions to wasp venom, as classified by the Mueller scale, with simple blood parameters such as EB/LR and EBP/LR. While these results are promising, they are limited by the small sample size. Further research involving a larger group of patients is necessary to better understand these observed relationships.

## Figures and Tables

**Table 1:** Hematological analysis of peripheral blood from patients in the Mueller scale III and IV group and the Mueller scale I and II group. Results are presented as mean ± SEM. Bold indicates parameters with significant differences.

	Mueller Grade III and IV		Mueller Grade I and II		*p* Value (Comparisons Between Different Mueller Scale Groups)	Control	
	Mean	SD	Mean	SD		Mean	SD
RBC [×10^12^/L]	4.83	0.10	4.74	0.12	0.775	4.87	0.63
HGB [g/dL]	14.20	0.29	14.03	0.41	0.679	14.71	1.88
HCT [%]	41.87	0.82	41.26	1.02	0.805	43.03	5.61
MCV [fL]	86.77	0.95	87.16	0.99	0.851	88.31	3.50
MCH [pg]	29.49	0.36	29.60	0.46	0.944	30.26	1.38
MCHC [g/dL]	33.99	0.17	33.99	0.26	0.503	34.26	0.76
PLT [×10^9^/L]	253.30	12.25	266.47	17.86	0.876	251.34	60.76
MPV [fL]	10.91	0.19	11.07	0.24	0.353	9.13	0.88
WBC [×10^9^/L]	6.46	0.32	7.12	0.36	0.617	6.09	1.74
LYMPH w.b. [×10^3^/µL]	2.12	0.11	1.97	0.12	0.333	1.64	0.62
LYMPH [%]	33.45	1.69	28.65	2.03	0.062	27.09	7.66
NEUT w.b. [×10^3^/µL]	3.60	0.30	4.42	0.37	0.073	3.76	1.33
NEUT [%]	54.27	2.07	60.92	2.46	0.035	61.41	8.80
MONO w.b. [×10^3^/µL]	0.54	0.03	0.55	0.04	0.862	0.40	0.11
MONO [%]	8.40	0.29	7.80	0.56	0.322	6.57	1.44
EO w.b. [×10^3^/µL]	0.20	0.03	0.15	0.02	0.131	0.17	0.15
EO [%]	3.24	0.48	2.16	0.33	0.056	2.81	2.28
BASO w.b. [×10^3^/µL]	0.04	0.00	0.03	0.00	0.189	0.04	0.02
BASO [%]	0.67	0.07	0.47	0.07	0.051	0.68	0.31

**Table 2:** Immune index calculations derived from peripheral blood cell counts in patients from the Mueller scale III and IV group, the Mueller scale I and II group, and control group. PLR—platelet-to-lymphocyte ratio; NLR—neutrophil-to-lymphocyte ratio; SII—systemic immune-inflammation index (SII = platelets × neutrophils/lymphocytes); SIRI—systemic inflammation response index (SIRI = monocytes × neutrophils/lymphocytes). Results are presented as mean ± SEM. Bold indicates parameters with significant changes.

	Mueller Grade III and IV		Mueller Grade I and II		Control Group		*p* Value Mueller III–IV vs. Mueller I–II	*p* Value Mueller III–IV vs. Control	*p* Value Mueller II–II vs. Control
	Mean	SEM	Mean	SEM	Mean	SEM			
PLR (P/L)	131.700	9.776	143.800	13.310	168.000	8.900	0.727	0.0131	0.0684
SII (P × N/L)	515.900	77.730	655.600	88.360	619.600	43.380	0.092	0.0254	0.8898
SIRI (N × M/L)	1.065	0.164	1.354	0.216	0.947	0.065	0.057	0.5117	0.0871
NRL (N/L)	0.678	0.059	0.507	0.056	2.540	0.153	0.310	0.0001	0.0001
E/L	0.093	0.012	0.075	0.010	0.078	0.007	0.183	0.1645	0.8239
B/L	0.021	0.002	0.018	0.002	0.024	0.001	0.343	0.2316	0.0279
**((E × B)/L) × 100**	**0.499**	**0.096**	**0.240**	**0.046**	**0.283**	**0.030**	**0.015**	**0.0463**	**0.3721**
(E × B × N)/L	0.017	0.004	0.010	0.002	0.011	0.001	0.218	0.1633	0.9288
P × E/L	23.220	3.078	19.960	2.675	19.300	1.896	0.520	0.2539	0.7525
P × B/L	5.599	0.836	4.729	0.777	5.860	0.438	0.452	0.4435	0.1156
**(E × B × P)/L**	**1.260**	**0.243**	**0.626**	**0.107**	**0.718**	**0.081**	**0.034**	**0.0388**	**0.633**
(N × M × E)/L	0.181	0.036	0.176	0.029	0.112	0.011	0.546	0.1325	0.0432
(N × M × E)/L	0.048	0.011	0.045	0.008	0.036	0.004	0.749	0.7659	0.525
N × M × E × B)/L	0.010	0.003	0.006	0.001	0.004	0.001	0.438	0.0613	0.3744

**Table 3:** Cytokine index calculations were derived from the concentrations of cytokines in serum and cell counts in the peripheral blood of patients from the Mueller scale III and IV group and the Mueller scale I and II group. Results are presented as mean ± SEM.

	Mueller Scale III and IV Group		Mueller Scale I and II Group		*p* Value
	Mean	SEM	Mean	SEM	
Th1/lymphocytes	577.43	260.81	584.08	235.30	0.981
Th2/lymphocytes	2413.08	1666.72	2041.60	1717.34	0.868
Th9/lymphocytes	115.72	17.57	107.85	17.62	0.666
Th17/lymphocytes	11.24	1.83	14.17	2.64	0.247
(Treg/lymphocytes)/10^3^	34.86	10.99	68.18	26.02	0.289
Th1/Th2	0.82	0.26	0.67	0.18	0.627
Th1/Th9	3.88	1.25	4.38	1.13	0.713
Th1/Th17	148.48	105.50	61.88	29.58	0.377
(Th1/Treg) × 10^3^	8.00	1.36	6.22	0.86	0.316
Th2/Th9	13.40	6.73	15.17	11.45	0.875
Th2/Th17	139.30	97.71	80.31	52.83	0.584
(Th2/Treg) × 10^3^	20.50	4.74	26.90	8.10	0.529
Th9/Th17	43.52	33.96	13.99	8.47	0.333
(Th9/Treg) × 10^3^	4.97	1.35	2.68	0.56	0.150
(Th17/Treg) × 10^3^	0.70	0.16	0.49	0.09	0.290

## Data Availability

The datasets generated during and/or analyzed during the current study are available from the corresponding author upon reasonable request.

## References

[1] Müller U. (1989). Insect sting allergy--clinical aspects, diagnosis and therapy. Wien. Med. Wochenschr..

[2] Banks B.E.C., Shipolini R.A. (1986). Chemistry and Pharmacology of Honey-bee Venom. Venoms of the Hymenoptera.

[3] Worm M., Moneret-Vautrin A., Scherer K., Lang R., Fernandez-Rivas M., Cardona V., Kowalski M.L., Jutel M., Poziomkowska-Gesicka I., Papadopoulos N.G. (2014). First European data from the network of severe allergic reactions (NORA). Allergy.

[4] Luo L., Kamau P.M., Lai R. (2022). Bioactive Peptides and Proteins from Wasp Venoms. Biomolecules.

[5] Nittner-Marszalska M., Kowal A., Szewczyk P., Guranski K., Ejma M. (2017). Wasp Venom Immunotherapy in a Patient with Immune-Mediated Inflammatory Central Nervous System Disease: Is it Safe?. J. Investig. Allergol. Clin. Immunol..

[6] Mueller U.R. (1990). Insect Sting Allergy, Clinical Picture, Diagnosis, and Treatment.

[7] Graif Y., Romano-Zelekha O., Livne I., Green M.S., Shohat T. (2009). Increased rate and greater severity of allergic reactions to insect sting among schoolchildren with atopic diseases. Pediatr. Allergy Immunol..

[8] Siracusa A., Folletti I., van Wijk R.G., Jeebhay M.F., Moscato G., Quirce S., Raulf M., Ruëff F., Walusiak-Skorupa J., Whitaker P. (2015). Occupational anaphylaxis--an EAACI task force consensus statement. Allergy.

[9] Feás X. (2021). Human Fatalities Caused by Hornet, Wasp and Bee Stings in Spain: Epidemiology at State and Sub-State Level from 1999 to 2018. Biology.

[10] Feás X., Vidal C., Remesar S. (2022). What We Know about Sting-Related Deaths? Human Fatalities Caused by Hornet, Wasp and Bee Stings in Europe (1994–2016). Biology.

[11] Ludman S.W., Boyle R.J. (2015). Stinging insect allergy: Current perspectives on venom immunotherapy. J. Asthma Allergy.

[12] Sturm G.J., Varga E.-M., Roberts G., Mosbech H., Bilò M.B., Akdis C.A., Antolín-Amérigo D., Cichocka-Jarosz E., Gawlik R., Jakob T. (2018). EAACI guidelines on allergen immunotherapy: Hymenoptera venom allergy. Allergy.

[13] Bonifazi F., Jutel M., Biló B.M., Birnbaum J., Muller U., EAACI Interest Group on Insect Venom Hypersensitivity (2005). Prevention and treatment of hymenoptera venom allergy: Guidelines for clinical practice. Allergy.

[14] Lawrence M.G., Woodfolk J.A., Schuyler A.J., Stillman L.C., Chapman M.D., Platts-Mills T.A.E. (2017). Half-life of IgE in serum and skin: Consequences for anti-IgE therapy in patients with allergic disease. J. Allergy Clin. Immunol..

[15] He Q., Wang S., Chen H., Long L., Xiao B., Hu K. (2023). The neutrophil-to-lymphocyte and monocyte-to-lymphocyte ratios are independently associated with clinical outcomes of viral encephalitis. Front. Neurol..

[16] Azab B., Camacho-Rivera M., Taioli E. (2014). Average Values and Racial Differences of Neutrophil Lymphocyte Ratio among a Nationally Representative Sample of United States Subjects. PLoS ONE.

[17] Demirkol S., Balta S., Cakar M., Unlu M., Arslan Z., Kucuk U. (2013). Red cell distribution width: A novel inflammatory marker in clinical practice. Cardiol. J..

[18] Yenigun A., Sezen S., Calim O.F., Ozturan O. (2016). Evaluation of the eosinophil-to-lymphocyte ratio in pediatric patients with allergic rhinitis. Am. J. Rhinol. Allergy.

[19] Jiang Y., Ma W. (2017). Assessment of Neutrophil-to-Lymphocyte Ratio and Platelet-to-Lymphocyte Ratio in Atopic Dermatitis Patients. Med. Sci. Monit..

[20] Dogru M., Citli R. (2017). The neutrophil-lymphocyte ratio in children with atopic dermatitis: A case-control study. Clin. Ter..

[21] Hagino T., Saeki H., Fujimoto E., Kanda N. (2023). The Eosinophil-to-Lymphocyte Ratio Acts as an Indicator for Improvement of Clinical Signs and Itch by Upadacitinib Treatment in Atopic Dermatitis. J. Clin. Med..

[22] Arwas N., Shvartzman S.U., Goldbart A., Bari R., Hazan I., Horev A., Tripto I.G. (2023). Elevated Neutrophil-to-Lymphocyte Ratio Is Associated with Severe Asthma Exacerbation in Children. J. Clin. Med..

[23] Shi G., Zhao J.W., Ming L. (2017). Clinical significance of peripheral blood neutrophil-lymphocyte ratio and plateletlymphocyte ratio in patients with asthma. J. South. Med. Univ..

[24] Szymański Ł., Urbańska W., Ciepielak M., Cios A., Stankiewicz W., Stelmasiak M., Rzeszotarska A., Korsak J., Lewicki S., Chciałowski A. (2022). Time-dependent effect of desensitization with wasp venom on selected parameters of the immune system. Sci. Rep..

[25] Urbańska W., Szymański L., Ciepelak M., Cios A., Stankiewicz W., Klimaszewska E., Lieto K., Skopek R., Chciałowski A., Lewicki S. (2023). Time-dependent cytokines changes in ultra-rush wasp venom immunotherapy. Sci. Rep..

[26] Matuszewski M., Szarpak L., Pruc M., Pedrycz-Wieczorska A., Kilic M., Ak R., Jankowski L., Bragazzi N.L., Moghadam M.G., Herman A.P. (2023). Platelet-to-lymphocyte ratio as a prognostic biomarker for COVID-19 severity: A single center retrospective data analysis and systematic review with meta-analysis of 187 studies. Disaster Emerg. Med. J..

[27] Li C., Tian W., Zhao F., Li M., Ye Q., Wei Y., Li T., Xie K. (2018). Systemic immune-inflammation index, SII, for prognosis of elderly patients with newly diagnosed tumors. Oncotarget.

[28] Dang H., Mao W., Wang S., Sha J., Lu M., Cong L., Meng X., Li H. (2023). Systemic inflammation response index as a prognostic predictor in patients with acute ischemic stroke: A propensity score matching analysis. Front. Neurol..

[29] Făgărășan I., Rusu A., Comșa H., Simu T.-D., Vulturar D.-M., Todea D.-A. (2023). IL-6 and Neutrophil/Lymphocyte Ratio as Markers of ICU Admittance in SARS-CoV-2 Patients with Diabetes. Int. J. Mol. Sci..

[30] Lee J., Lozano-Ruiz B., Yang F.M., Fan D.D., Shen L., González-Navajas J.M. (2021). The Multifaceted Role of Th1, Th9, and Th17 Cells in Immune Checkpoint Inhibition Therapy. Front. Immunol..

[31] Nahm F.S. (2022). Receiver Operating Characteristic Curve: Overview and Practical Use for Clinicians. Korean J. Anesthesiol..

[32] Tahir N., Zahra F. (2024). Neutrophilia. StatPearls.

[33] Rosales C. (2018). Neutrophil: A Cell with Many Roles in Inflammation or Several Cell Types?. Front. Physiol..

[34] Day J.H., Buckeridge D.L., Welsh A.C. (1994). Risk assessment in determining systemic reactivity to honeybee stings in sting-threatened individuals. J. Allergy Clin. Immunol..

[35] Hollstein M.M., Matzke S.S., Lorbeer L., Forkel S., Fuchs T., Lex C., Buhl T. (2022). Intracutaneous Skin Tests and Serum IgE Levels Cannot Predict the Grade of Anaphylaxis in Patients with Insect Venom Allergies. J. Asthma Allergy.

[36] Wilson A.B., Deighton J., Lachmann P.J., Ewan P.W. (1994). A comparative study of IgG subclass antibodies in patients allergic to wasp or bee venom. Allergy.

[37] Reisman R.E., DeMasi J.M. (1989). Relationship of serum venom-specific IgE titers to clinical aspects of stinging insect allergy. Int. Arch. Allergy Appl. Immunol..

[38] Kopač P., Custovic A., Zidarn M., Šilar M., Šelb J., Bajrović N., Eržen R., Košnik M., Korošec P. (2021). Biomarkers of the Severity of Honeybee Sting Reactions and the Severity and Threshold of Systemic Adverse Events During Immunotherapy. J. Allergy Clin. Immunol. Pract..

[39] Nittner-Marszalska M., Małolepszy J., Medrala W. (1993). Evaluation of diagnostic value of skin test, venom specific antibodies against EgE and basophil histamine release test in hymenoptera allergy. Pneumonol. Alergol. Pol..

[40] Warrington R. (2006). Lack of Correlation between Severity of Clinical Symptoms, Skin Test Reactivity, and Radioallergosorbent Test Results in Venom-Allergic Patients. Allergy Asthma Clin. Immunol..

[41] Sturm G.J., Heinemann A., Schuster C., Wiednig M., Groselj-Strele A., Sturm E.M., Aberer W. (2007). Influence of total IgE levels on the severity of sting reactions in Hymenoptera venom allergy. Allergy.

[42] Van der Linden P.-W.G., Hack C.E., Poortman J., Vivié-Kipp Y.C., Struyvenberg A., van der Zwan J.K. (1992). Insect-sting challenge in 138 patients: Relation between clinical severity of anaphylaxis and mast cell activation. J. Allergy Clin. Immunol..

[43] Tomsitz D., Brockow K. (2017). Component Resolved Diagnosis in Hymenoptera Anaphylaxis. Curr. Allergy Asthma Rep..

[44] Bilò M.B., Ollert M., Blank S. (2019). The role of component-resolved diagnosis in Hymenoptera venom allergy. Curr. Opin. Allergy Clin. Immunol..

[45] Guan K., Li L.-S., Yin J. (2016). Use of sIgE/T-IgE in Predicting Systemic Reactions: Retrospective Analysis of 54 Honeybee Venom Allergy Cases in North China. Chin. Med. J..

[46] Hamilton R.G., Williams P.B., Specific IgE Testing Task Force of the American Academy of Allergy, Asthma & Immunology, American College of Allergy, Asthma and Immunology (2010). Human IgE antibody serology: A primer for the practicing North American allergist/immunologist. J. Allergy Clin. Immunol..

[47] Gattinger P., Lupinek C., Kalogiros L., Silar M., Zidarn M., Korosec P., Koessler C., Novak N., Valenta R., Mittermann I. (2018). The culprit insect but not severity of allergic reactions to bee and wasp venom can be determined by molecular diagnosis. PLoS ONE.

[48] Zhai G., Wang J., Liu Y., Zhou Y. (2021). Platelet-lymphocyte ratio as a new predictor of in-hospital mortality in cardiac intensive care unit patients. Sci. Rep..

[49] Wawryk-Gawda E., Żybowska M., Ostrowicz K. (2023). The Neutrophil to Lymphocyte Ratio in Children with Bronchial Asthma. J. Clin. Med..

[50] Cansever M., Sari N. (2022). The association of allergic rhinitis severity with neutrophil-lymphocyte and platelet-lymphocyte ratio in children. North. Clin. Istanb..

[51] Wang S., Pan X., Jia B., Chen S. (2023). Exploring the Correlation Between the Systemic Immune Inflammation Index (SII), Systemic Inflammatory Response Index (SIRI), and Type 2 Diabetic Retinopathy. Diabetes Metab. Syndr. Obes..

[52] Bayraktar M.F., Coşgun M. (2024). Can the systemic immune inflammation index (SII) and the systemic inflammation response index (SIRI) predict the severity of coronary artery disease?. Northwestern Med. J..

[53] Ma R., Cui L., Cai J., Yang N., Wang Y., Chen Q., Chen W., Peng C., Qin H., Ding Y. (2024). Association between systemic immune inflammation index systemic inflammation response index adult psoriasis: Evidence from NHANES. Front. Immunol..

[54] Yin X., Zhang Y., Zou J., Yang J. (2024). Association of the systemic immune-inflammation index with all-cause and cardiovascular mortality in individuals with rheumatoid arthritis. Sci. Rep..

[55] Tian T., Xie M., Sun G. (2024). Association of systemic immune-inflammation index with asthma and asthma-related events: A cross-sectional NHANES-based study. Front. Med..

[56] Zeng-Yun-Ou Z., Zhong-Yu J., Wei L. (2022). Bidirectional associations between eosinophils, basophils, and lymphocytes with atopic dermatitis: A multivariable Mendelian randomization study. Front. Immunol..

